# Lipid-Based Drug Delivery Systems for Diseases Managements

**DOI:** 10.3390/biomedicines10092137

**Published:** 2022-08-31

**Authors:** Douweh Leyla Gbian, Abdelwahab Omri

**Affiliations:** Department of Chemistry and Biochemistry, The Novel Drug and Vaccine Delivery Systems Facility, Laurentian University, Sudbury, ON P3E 2C6, Canada

**Keywords:** liposomes, drug delivery, characterization, applications

## Abstract

Liposomes are tiny lipid-based vesicles composed of one or more lipid bilayers, which facilitate the encapsulation of hydrophilic, lipophilic, and amphiphilic biological active agents. The description of the physicochemical properties, formulation methods, characteristics, mechanisms of action, and large-scale manufacturing of liposomes as delivery systems are deeply discussed. The benefits, toxicity, and limitations of the use of liposomes in pharmacotherapeutics including in diagnostics, brain targeting, eye and cancer diseases, and in infections are provided. The experimental approaches that may reduce, or even bypass, the use of liposomal drug drawbacks is described. The application of liposomes in the treatment of numerous diseases is discussed.

## 1. Introduction

For the past decades, liposomes have been increasingly used for their applications in numerous fields including cancer therapy, infectious diseases, and gene delivery. They are one of the most versatile carriers owing to their ability to encapsulate, protect, and transport molecules with various chemical and physical properties [1]. Liposomes are microscopic versatile lipid-based vesicles consisting of one or more lipid bilayers in which hydrophilic, lipophilic, and amphiphilic compounds can be encapsulated [2,3]. Indeed, hydrophilic drugs can be encapsulated in the aqueous layer, hydrophobic drugs are added in the lipid bilayer, and amphiphilic compounds are incorporated at the bilayer interface [4]. They are the most widely used nanoparticles for drug delivery and increase biocompatibility, bioavailability, and therapeutic index, improve site-specific drug delivery, are biodegradable, and reduce the toxicity of encapsulated drugs [5,6,7]. Due to their submicron size, they also show good vascular and tissue penetration [8]. This review aims to discuss the methods of production and characterization of liposomes and their different applications in the pharmaceutical industry.

## 2. Composition of Liposomes

Liposomes are mostly synthesized with cholesterol and an array of phospholipids constituents, whose chemical properties ultimately dictate liposomal properties [9]. The most commonly used phospholipids are phosphatidylcholines (PC), phosphatidylethanolamine (PE), phosphatidylserines (PS), phosphatidylglycerol (PG), and sphingomyelin [10]. Liposomal net charges vary depending on the charges of phospholipids and can be neutral, cationic, or anionic [4]. Since both positively and negatively charged liposomes tend to be cleared faster due to their interactions with opsonizing proteins, liposomes with neutral charges are more commonly used [11]. The physicochemical properties of liposomes, which include size, charge, surface properties and encapsulation efficiency, can greatly affect their stability and activity in vivo [4]. For instance, liposomes made of unsaturated egg or soybean PC are usually highly permeable and less stable, whereas saturated phospholipids with long acyl chains such as 1,2-dipalmitoyl-sn-glycero-3-phosphatidylcholine (DPPC) give more rigid and stable liposomes with an impermeable bilayer [12].

## 3. Classification of Liposomes

**Figure 1:**
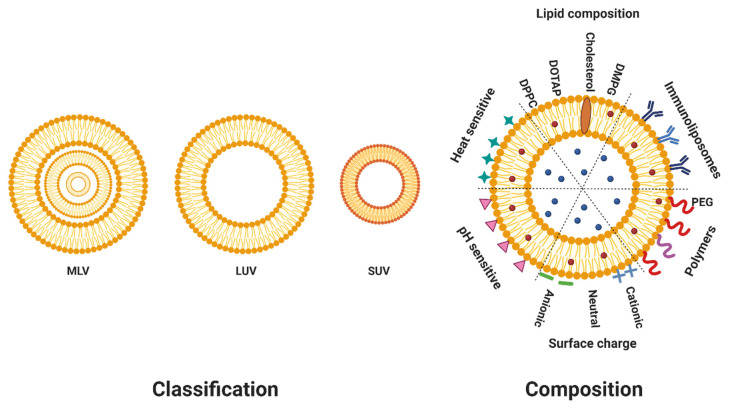
Classification and composition of liposomes. Liposomes can be classified based on their size, their lamellarity, and their composition. The red dots represent hydrophobic drugs whereas the blue ones represent hydrophilic drugs.

Based on their size and number of lipid bilayers, liposomes can be classified into two main categories (Figure 1): multilamellar vesicles (MLVs) (>500 nm), which consist of more than one bilayer, arranged in an onion structure of phospholipid spheres separated by water, and unilamellar vesicles made of one bilayer [13]. Unilamellar vesicles can be further classified into large unilamellar vesicles (LUVs) (>100 nm) and small unilamellar vesicles (SUVs) (20–100 nm) [14]. The size of liposomes can affect their circulation half-life. In fact, liposomes larger than 100 nm are more prone to opsonization and clearance by the reticuloendothelial system [15]. Furthermore, both size and the number of lipid bilayers affect the amount of drug encapsulated in liposomes [13].

## 4. Methods of Preparation of Liposomal Therapeutics

**Figure 2:**
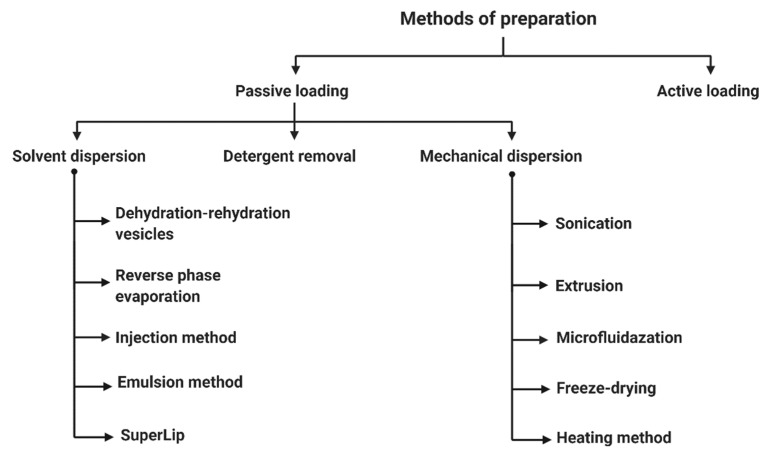
Methods of liposomes preparation.

Liposomal preparation techniques can be divided in two main groups: active and passive loading techniques, as shown in Figure 2. Active encapsulation includes methods that incorporate drugs after liposome preparation. They are mostly gradient loading techniques involving buffer or ammonium sulfate gradients [16]. On the other hand, passive loading techniques include methods where a drug is encapsulated during the formation of liposomes [12]. These include new and conventional liposomes preparation, which all consist of a few basic steps: the dissolution of lipids in an organic phase, subsequent dehydration of the organic phase, the dispersion of the lipid layer in an aqueous solution, and the purification and analysis of the resulting liposomes [13,17]. They are still very popular and preferred because of their simplicity [7]. However, they do not translate well in the scale-up of liposomes for industrial production.

### 4.1. Passive Loading Techniques

#### 4.1.1. Thin Film Hydration and Dehydration–Rehydration Vesicles (DRV) Methods

The thin film hydration or Bangham method is one of the simplest and most used methods to prepare liposomes [18,19]. It involves the dissolution of phospholipids in an organic solvent such as chloroform in a flask, followed by the evaporation of the solvent under high vacuum to form a thin lipid layer on the walls of the flask [10]. The lipid film is subsequently rehydrated with an aqueous solution in which a hydrophilic drug of choice was previously dissolved and mechanical agitation or vortexing is applied to detach the layer [20]. Lipophilic drugs on the other hand are directly dissolved in the organic solvents along with the phospholipids. Phospholipids will self-assemble into bilayers around the solution, forming liposomes. Liposomes produced with this method are 10–100 µm MLVs, not small enough for further applications, and as such it is usually combined with sonication or extrusion [21]. The DRV method includes the same first steps as the thin film hydration method; however, after the formation of liposomes, sonication or extrusion are applied to reduce the size of the vesicles. The obtained formulation is then freeze-dried, and the resulting powder is rehydrated when needed. Encapsulation efficiencies (EEs) with those methods can go up to 90% with lipophilic compounds, but is around 10–30% with hydrophilic drugs [9,14]. EE can be increased by the active drug loading technique. The major drawbacks of the methods are low entrapment efficiency, residual organic solvent and small scale production [18].

#### 4.1.2. Reverse Phase Evaporation Method (RPE)

RPE is carried out by dissolving phospholipids in an organic solvent (diethyl ether or isopropyl ether), adding small amounts of the aqueous phase in organic solvent, forming a water-in-oil emulsion and sonicating the solution to produce inverted micelles [18]. This step is then followed by the removal of the organic solvent by rotary evaporation, generating a viscous gel. The gel later collapses and the excess phospholipids in the environment form a second layer around micelles, resulting in the formation of liposomes [10]. The contact between the organic solvent and the drug to be encapsulated can degrade fragile molecules such as peptides, making RPE unsuitable for their encapsulation [18]. The large aqueous space created in liposomes with this method enables the encapsulation of macromolecules, with EE going up to 90% with some compounds [22].

#### 4.1.3. Injection Method

In this method, phospholipids are dissolved in ethanol or ether and the lipid solution is injected dropwise with a needle into an aqueous solution containing a hydrophilic drug. Unilamellar liposomal vesicles are then formed [23]. The major drawbacks of this technique include its time-consuming process, a high organic residue in the final suspension, and difficulty removing ethanol since it forms an azeotropic mixture with water [24]. Furthermore, an evaporation could damage the structure of vesicles and the particle size distribution is difficult to control with this method [21]. Additionally, since lipids are poorly soluble in ethanol, ethanol injection often leads to the formation of heterogeneous liposomes [24]. EE reported for the injection method is around 20% for hydrophilic compounds and greater than 60% for some lipophilic compounds. The recently developed inkjet method is a modern variation of the injection method that offers excellent control of particle size and potential for scaling up. An inkjet printer is used to inject a phospholipids solution into water, generating SUVs (20–100 nm) with higher EE than thin film hydration and high reproducibility [21].

#### 4.1.4. Emulsion Method

For this method phospholipids are dissolved in a water/organic solvent mixture, forming a water-in-oil (W/O) emulsion [25]. The mixture is then transferred into a large aqueous solution and vigorously agitated, producing a double emulsion, a water-in-oil-in-water emulsion (W/O/W) [26]. A second lipid monolayer surrounds the particle, forming droplets with an aqueous core and a lipid bilayer containing residual organic solvent [10]. The organic solvent can be removed by passing some nitrogen through the double emulsion, resulting in the formation of unilamellar liposomes with high EE. An advantage of this technique includes the control over liposomes’ size by agitation and the amount of lipids used.

#### 4.1.5. Detergent Removal Method

Here, detergent-lipids micelles are formed by either hydrating phospholipids with a solution of detergent or drying phospholipids and detergent from an organic solution and hydrating them with an aqueous solution [25]. The detergent is then removed from the micellar solution by dilution, dialysis, column chromatography, or adsorption, resulting in the formation of liposomes. This method is, however, time-consuming, leads to low EE of hydrophobic drugs, detergent residues in the formulations, and even methods employed for the removal of detergent can also remove small hydrophilic compounds [25].

Mechanical methods are often used for post-formation processing of liposomes to disrupt LUVs and modify their size, lamellarity, or homogeneity to ultimately form SUVs.

#### 4.1.6. Sonication

This is the most widely used method for the preparation of SUVs. MLVs are sonicated in a bath sonicator or with a probe sonicator [15]. In the former, the liposome dispersion in a container is placed into a bath sonicator, which allows for a better control of the temperature and the protection of liposomes in a sterile vessel. On the other hand, in a probe sonication, the tip of the sonicator is directly inserted into the liposome dispersion, where the energy input is very high and results in local heat [27]. There is also a risk of metal sloughing off and contaminating the solution. The main inconvenience of this method includes low internal volume, possible degradation of drugs and phospholipids, possible contamination (probe sonication), and a heterogeneous mix of MLVs and SUVs [13].

#### 4.1.7. Membrane Extrusion

The extrusion method involves the size reduction of previously prepared liposomes by passing them through polycarbonate filters of different pore size (depending on the application) with repeated cycles of extrusion under moderate-high pressure [25]. The process can be time-consuming [15].

#### 4.1.8. Microfluidization Method

In this process, a lipid solution in organic solvent is injected into a central feeding channel while aqueous solutions are added to side channels, which intersect with the main channel at the center [10]. As the lipid solution passes between two aqueous streams in the microfluidic channel, mixing occurs and thus monodisperse nanometric liposomes are obtained in the outer channels [28,29]. The size of the liposomes can be controlled by adjusting the flow conditions, so no size reduction is needed post preparation [18]. Microfluidization suffers from high solvent residues in liposome suspensions and a difficulty to reach high production scale. Additionally, the production cost of the micro-circuit used in this method is higher than other conventional methods [10,30].

#### 4.1.9. Freeze-Drying Method

The freeze-drying method is the most popular technique used to stabilize liposomes and extend their storage. It involves freezing liposomes-cryoprotectant mixtures at −40 °C for some time and then drying them at the same temperature for 48 h, followed by drying of the product at room temperature [31,32]. Upon the addition of an aqueous solution, the lyophilized powder spontaneously forms spherical lipid vesicles. Sucrose and glycerol (cryoprotectants) were shown to stabilize liposomes during this process by preventing the aggregation and fusion of mini-domains [31]. Liposomes made with this method have an average size of 100–300 nm and an EE of 40–60%.

#### 4.1.10. Heating Method

In this method developed by Mozafari, phospholipids are added to an aqueous solution containing glycerol (3% v/v) at a bath temperature up to 120 °C [33]. The use of glycerol is motivated by its solubility in water and its ability to act as an isotonic agent, increasing the stability of liposomes. It is also a physiologically acceptable material which does not need to be removed from the final product, reducing toxicity of liposomes [17]. Materials can be encapsulated at any step of the process except for heat-sensitive compounds (e.g., DNA), which should be added at ambient temperature, after liposome preparation [33]. Liposomes produced with this technique have nanometric sizes, long-term stability (12–14 months), and low EE that can however go up to 81% with DNA [33]. The main advantage of this technique is that it does not use any organic solvent for the preparation of liposomes [27]. Additionally, the use of heat to prepare liposomes abrogates the need to carry out a sterilization step afterwards [17].

#### 4.1.11. SuperLip Method

Finally, Supercritical assisted liposomes formation (SuperLip) is an emerging alternative to traditional techniques [34]. Unlike other methods, this process begins with the creation of water droplets through a spray atomization. The droplets are passed into a high-pressure vessel filled with phospholipids dissolved in an expanded liquid (ethanol and carbon dioxide) [35]. The droplets are quickly surrounded by phospholipids, creating lipid vesicles. Liposomes aqueous suspensions are collected from the bottom of the vessel [36]. The expanded liquid is then removed from the top of the vessel, and depressurized to separate carbon dioxide from ethanol [35]. The advantages of the method are its simple continuous operative layout not requiring numerous steps, its lower solvent residue and its high reproducibility and encapsulation efficiency (up to 99% for hydrophilic compounds and 87% for lipophilic compounds) [34]. This method shows potential for long circulating drug delivery systems, obtention of Nanometric dimensions, minimal solvent residue, high Encapsulation efficiencies, and no variations between batch production as compared with the previously described methods.

### 4.2. Active Encapsulation

Active encapsulation or remote drug loading works with drugs that are fairly soluble and able to go from uncharged species (freely diffusible across the lipid membrane) to charged ones (not freely diffusible across the lipid membrane), and it is therefore limited to amphipathic weak acids or bases [37]. Non-amphipathic drugs can however be chemically modified in some cases with cyclodextrins, for instance [38,39]. The driving force of this process is the transmembrane gradient of ions, as they can be exchanged with amphipathic drugs inside liposomes [38]. This gradient is created by concentrations of (NH_4_)_2_SO_4_ or Ca(C_2_H_3_O_2_)_2_ in the liposomes, which are 1000-fold greater than concentrations outside the liposome. Indeed, remote loading uses an ammonium sulfate gradient to encapsulate weak bases into liposomes or a calcium acetate gradient for weak acids [39]. The un-ionized drug-base or drug-acid outside the liposomes crosses the liposomal membrane where it is trapped inside by its ionization and the formation of an insoluble salt with the intra-liposome counter ion [37]. A pH gradient can also be used for this technique [16]. Active encapsulation usually results in high EE and enables controlled drug release [38]. Table 1 below summarizes the advantages and disadvantages of the different preparation methods.

## 5. Methods of Characterization

Liposomes’ properties are highly dependent on their size, lamellarity, surface charges, and encapsulation efficiency [30]. Therefore, an accurate estimation of those parameters is vital. The concentration of drugs encapsulated can be determined using SpectraMax M5 plate reader, measuring absorbances and standard curve of known concentrations [41].

### 5.1. Particle Size

Dynamic light scattering (DLS) is the most used method to determine liposomes’ size in the sub-micron range by using light scattering from a laser that passes through liposomal solution to analyze the intensity of scattered light as a function of time. The Brownian motion of the particles correlates with their hydrodynamic diameter, which affects their scattering capacity [42]. DLS can also be used to study the stability of formulations over time, as particle sizes increase when the particles agglomerate, a sign of instability [29,43]. However, it does not provide accurate measurements for highly polydisperse samples and assumes a spherical shape for the liposomes [44]. Alternatively, electron microscopy techniques are powerful tools to study the size, size distribution, lamellarity, and shape of liposomes [40]. They are, however, expensive, time consuming, as the analysis is done manually and therefore prone to human error [42]. The combination of those two methods overcomes most of their shortcomings as stand-alone techniques and can provide a more accurate estimation of liposomal parameters. Asymmetric field flow fractionation has been increasingly used in recent years to measure the size distribution of polydisperse liposomal samples in the sub-micron range. Unfortunately, the high cost associated with the technique limits its widespread application [40]. Finally, laser diffraction and nanoparticle tracking analysis are commonly used, along with the novel multispectral advanced nanoparticle tracking analysis [44].

### 5.2. Lamellarity

Lamellarity is estimated with ^31^P NMR (nuclear magnetic resonance), where the degree of lamellarity is determined from the signal ratio before and after Mn^2+^ addition. This method is very dependent on Mn^2+^ and buffer concentrations and the type of liposome analysed [40]. Small angle X-ray scattering (SAXS) and electron microscopy are also used to determine lamellarity [45].

### 5.3. Zeta Potential

Zeta potential is an important factor used to approximate liposomes’ surface charge, which strongly affects their stability, pharmacokinetics properties, and affinity with entrapped drugs [20]. For instance, charged liposomes are less prone to aggregation while negatively charged liposomes improve stability and aggregation and are less prone to phagocytosis than positively charged ones [19]. Zeta potential is measured by the electrophoretic mobility of charged particles once an electric field is applied. It reflects the potential difference between the electric double layer of mobile particles and the layer of dispersant around them [43]. Adsorption of ions onto liposomes’ surfaces can affect their zeta potential and even their sign [46].

### 5.4. Encapsulation Efficiency (EE)

EE is defined as the percentage of drug successfully entrapped into liposomes with regards to the initial amount of drug used. EE mostly depends on phospholipids compositions, lipids to drug ratio, and the methods of preparation [9]. Various analytical methods like Ultraviolet-visible spectroscopy, high-pressure liquid chromatography, gas chromatography, gas chromatography-mass spectroscopy, and even quantitative NMR can be used to determine the concentration of drug inside the liposomes, depending on the nature of the active compound [27,47]. In an interesting study Peretz and coworkers used a cryo-electron microscopy technique to determine the exact concentration of drug encapsulated per liposome by calculating the trapped volume of individual liposomes using their micrographs [42]. This method can prove extremely useful in the development of liposomal pharmaceutics.

## 6. Stabilization of Liposomes

The main parameters affecting liposomes stability are size, composition, and electric charge. Stability issues are also caused by chemical hydrolysis of acyl ester bonds and oxidation of the polyunsaturated acyl chains, leading to drug leakage, fusion, and aggregation of liposomes [48]. Cholesterol plays an essential role in stabilizing liposomes and minimizing phospholipids exchange. It prolongs their circulation time by several hours. In addition, cholesterol is incorporated into phospholipids bilayers because it was shown to increase separation between the choline head groups and reduce the hydrogen bonding strengths and electrostatic interactions [46], thus making the lipid bilayer more stable and lowering its permeability to water and other molecules [9]. Polymers have been extensively used to stabilize liposomes, especially polyethylene glycol (PEG). PEG is indeed known to improve liposomes’ stability and enhance their circulation time by creating a protective hydrophilic layer on the surface of liposomes, hampering binding with opsonin protein and serum protein-mediated lipid elimination [48,49]. It is also possible to increase stability through cross-linking the hydrophobic tail groups of lipids within lipid bilayers or the functionalized headgroups of adjacent lipid bilayer of multilamellar liposomes.

Since the aqueous environment in which liposomes exist is propitious to the stability issues previously mentioned, freeze-drying offers a possibility to overcome these issues [48]. Freeze-drying, discussed above, is one of the most used post-processing techniques to increase the stability of liposomes, as it eliminates water molecules and prevents the degradation of sensitive substances [50]. It is, however, necessary to develop new stabilization methods that do not involve surface modifications of liposomes to maintain their surface properties and prevent unwanted bio-interference interactions. Chen and coworkers offer a new method to stabilize liposomes using a stiff nanobowl in the aqueous layer. Indeed, for the first time they developed liposomal doxorubicin supported by nanobowls that show reduced drug leakage, improved drug delivery to tumor sites, and increased killing of tumor cells compared to conventional liposomes [51]. Similar methods that aim to support or stabilize the aqueous layer instead of the lipid layer should be explored in the future as they could improve drug loading and reduce leakage.

## 7. Scale-Up of Liposomes

The production of liposomes on a large scale in the pharmaceutical industry can be arduous, limiting their translation from bench to bedside. This is because of the limited feasibility of scaling most conventional bench-scale techniques to clinical scale production and the multi-step process of manufacturing them [52]. Other issues with scale-up include multi-step processes, non-uniformity in size distribution of the liposomes from batch to batch, exposure to high concentrations of organic solvents (degrade biological molecules), and low reproducibility. Therefore, new rapid, cost-effective, scalable manufacturing processes with reduced steps are needed. Microfluidics has been reported to be a cost-effective, scale-independent technique to include in the production process, offering high-throughput, continuous production with good reproducibility and particle size control [52,53]. It can eliminate the need for lipid hydration and extrusion since the vesicles are formed and hydrated in the microfluidic chamber [14]. Roces and coworkers demonstrated the production of SUVs PEGylated tumor-targeting liposomes with a high encapsulation efficiency (>90%) using microfluidics [52]. Furthermore, in a new technique called the nanoprecipitation/antisolvent technique, phospholipids were dissolved in a biocompatible solvent along with a stabilizer to spontaneously form sub-micron vesicles upon exposure to water. When optimized, this technology could eliminate the need for an extrusion and a filtration step, avoiding the use of organic solvents altogether [14]. There are still some challenges and issues of liposomes scale-up production that should be addressed, including oxidative degradation of liposomes, validation of depyrogenation of raw lipid materials, complete removal of residual traces of organic solvents, and batch uniformity.

## 8. Mode of Action

**Figure 3:**
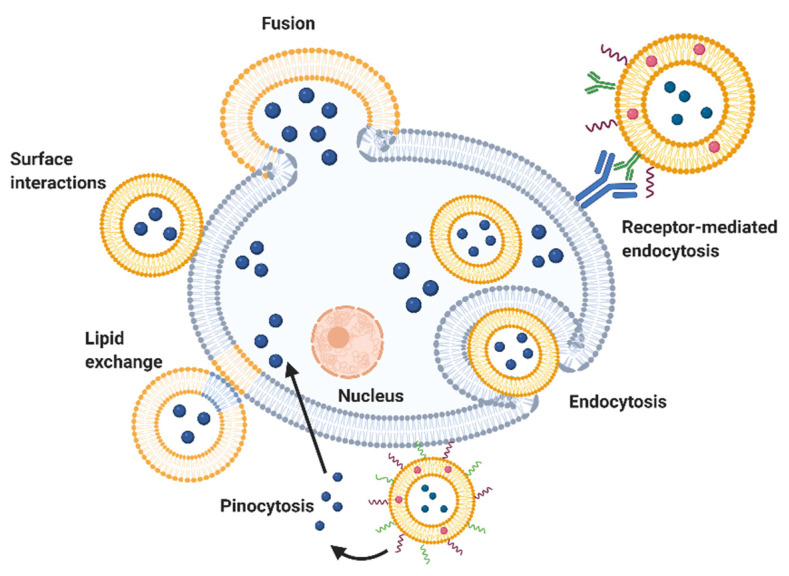
Mechanisms of liposomes uptake into cells. Liposomes can deliver their content into cells through various mechanisms including lipid exchange, surface interactions, fusion, endocytosis, and pinocytosis.

With the recent advent of surface-modified liposomes (Figure 3), it is possible to decorate the external surface of liposomes with various materials such as peptides, antibodies, polymers, pH-sensitive compounds, etc. Those liposomes can have specific bindings to cell receptors that create long-lasting drug release [26]. Long-circulating liposomes can be prepared by coating their surface with polyethylene glycol (PEG) [20]. PEG was indeed reported to increase liposomes stability, distribution in target tissues and their half-lives [22]. Additionally, pH-sensitive liposomes can direct the accumulation and the release of their content at a specific target site, such as cancer cells, which have been shown to be slightly acidic (pH 6.4–6.8 vs. normal pH 7.4). The design of pH-sensitive liposomes can be achieved with pH labile linkages, pH cleavable crosslinking, or by adding charge shifting polymers at their surface [54].

The similarity of liposomes with cell membranes and their small sizes make them particularly useful for intracellular drug delivery [23]. Liposomes can in fact interact with cells and deliver their material by four main mechanisms (Figure 3): (a) specific interactions with cell-surface components, (b) endocytosis by phagocytic cells, (c) fusion with cell membrane by insertion of lipids from liposomes into plasma membrane, followed by the release of liposomal content into the cytoplasm, and finally (d) exchange (swap) of liposomes’ bilayer components with cell membrane components [25]. More than one of these mechanisms can be operating at the same time. Neutral, anionic, and stimuli-responsive liposomes are internalized into the cells by endocytosis, releasing their contents into the cytoplasm [55]. On the other hand, cationic liposomes are reported to use membrane fusion and endocytosis to deliver drugs to cells [56]. PEGylated liposomes gradually release their content into the extracellular fluid (Figure 3), which then enters cells either via diffusion or pinocytosis. Finally, ligand-targeted liposomes enter into the cell via receptor-mediated endocytosis where ligands are removed and liposomes release their drug content [57]. Those various mechanisms are summarized in Figure 3 for clarity.

To overcome the fast elimination problem, immunoliposomes (surface modified liposomes programmed to be digested by macrophages, which then transferred their contents to target tissues, exploiting the immune response) show good promise [20].

## 9. Routes of Administration and Biodistribution

Liposomes can be administered via different routes, namely oral, topical, nasal, transdermal routes, and in the brain owing to their versatility [58]. They are chemically degraded and excreted through their uptake and clearance by the reticuloendothelial system (RES). Liposomes modify tissue distribution and the clearance of the loaded drugs. The physicochemical properties of the liposomes formed, such as the lipid composition, the surface charge, size, and route of administration, directly affect the pharmacokinetics of the liposomal drug. The main sites of accumulation of liposomes are tumor sites, liver, and spleen [59]. It is important to note that the pharmacokinetics of the liposome-drug depends on the carrier and not the encapsulated drug until the drug is released [60].

Once in the bloodstream, plasma proteins, such as opsonins will help the RES recognize and eliminate liposomes. This causes lipid transfers and rearrangements in liposomes, which induces liposome breakdown and rapid release of the cargo to the plasma [61]. The encapsulated drug will then interact with its target cells [60]. Finally, liposomes are eliminated by the target tissues after recognition by the RES at the hepatic level and metabolized by Kupffer cells. They can also be metabolized by macrophages in the spleen [59].

## 10. Advantages and Toxicity

Liposomes have several advantages compared to free drugs or other delivery systems. They offer a reduced toxicity, reduced degradation of drugs, and a high biocompatibility since they are made of naturally occurring phospholipids [41,62]. They can also increase the therapeutic index of drugs, offer direct targeting of specific tissues or receptors, and bypass multidrug resistance observed in cancer therapy and in antimicrobial therapy exhibited by bacteria [63].

In spite of all the advantages they provide liposomes still suffer from poor stability, low encapsulation of hydrophilic drugs, fast elimination in vivo, sterility issues, possible leakage of antibiotics, and the difficulty of scaling up [5,64]. Finally, organic solvents from conventional methods of preparation can be toxic to humans and could potentially degrade encapsulated active agents [40]. Liposomes may even enter healthy tissue and cause cytotoxicity and an immune reaction [63].

## 11. Applications and Approved Treatments

In recent years, a great number of liposomal formulations have been developed to treat various diseases due their excellent drug encapsulation, which enhances the therapeutic efficacy of the drugs and reduces their associated toxicity. The versatility of liposomes makes their applications in various fields possible. Liposomes have been successfully developed for parenteral, transdermal, and oral drug delivery in a number of fields, namely eye and respiratory disorders, brain targeting, vaccine adjuvants, antimicrobial and cancer therapies, and even gene delivery [26]. The following Table 2 presents some liposomal formulations approved in various fields.

### 11.1. Liposomes in Diagnostic

Peptide-targeting liposomes tagged with imaging agents can be used to efficiently deliver diagnostic agents to targeted sites. They can be used for diagnostic purposes to detect several diseases, including cancer [76]. Song and coworkers constructed new dual-targeted paramagnetic liposomes for cancer diagnostic, with ανβ3-integrin and neuropilin-1 receptors on the surface and the MRI (magnetic resonance imaging) contrast agent Gd-DTPA inside the liposomes. The liposomal complexes were able to efficiently bind to tumor tissue and could potentially improve the effect of MRI contrast agents for tumor-specific imaging early on [77]. A recent study developed an approach in which reagent-loaded liposomes are used to effectively diagnose a SARS-CoV-2 gene target, even in cases missed by RT-qPCR [78]. This is a great tool, much needed to support coronavirus disease 2019 (COVID-19) detection.

### 11.2. Liposomes for Brain Targeting

There is a crucial need to enhance treatment of central nervous system (CNS) diseases such as multiple sclerosis, stroke, neurodegeneration, and brain tumors. CNS drug delivery has not been completely efficient mainly because of the blood–brain barrier, which serves as a selective semipermeable layer causing low drug permeability [79]. Due to their nature, liposomes have shown great promise in delivering therapeutics to the brain. Cationic liposomes are the most effective for drug delivery to the brain, probably because of their electrostatic interactions with the negatively charged cell membranes, which enhances nanoparticle uptake. However, due to their non-specific uptake and their binding to serum proteins, large amounts are needed for a therapeutic effect, which can be toxic [79]. It is necessary to develop stimuli-sensitive liposomes and liposomes with better targeting of specific brain areas.

### 11.3. Liposomes as Vaccine Adjuvants

Cationic liposomes are more potent carriers for vaccines, because they induce stronger immune reactions at low dose. Antigen–liposomal complexes can increase and maintain exposure of the antigen in lymph nodes, making for an enhanced uptake by immature phagocytic antigen-presenting cells such as macrophages and dendritic cells [63]. Recently, the global pandemic caused by the respiratory illness designated coronavirus disease 2019 (COVID-19) created an urgent international need for vaccines [74]. Moderna developed a pegylated liposomal mRNA vaccine (mRNA 1273), which encapsulates nucleoside-modified mRNA that encodes the SARS-CoV-2 spike (S) glycoprotein, with 94.1% effectiveness in a Phase 3 trial [75,80]. The vaccine is now widely administered in the USA and Canada for the prevention of COVID-19 since December 2020, with mild to moderate side effects reported in general [81].

### 11.4. Liposomes in Eye Disease

The development of ophthalmic drug delivery systems is very challenging because of the various ocular protection mechanisms against foreign substances, impeding drug entry (tear dilution, lacrimation) [82]. The eye can be divided into an anterior segment composed of structures such as the cornea, conjunctiva, iris, ciliary body, the lens and aqueous humor, and the posterior segment made of the retina, optic nerve, choroid, and vitreous humor. Some of the pathologies that can affect the anterior segment are dry eye disease, cataracts, conjunctivitis, and keratitis, while the posterior segment is affected by diseases causing significant damages in vision (irreversible blindness, glaucoma, and diabetic retinopathy). López-Cano and coworkers offer an extremely detailed review of liposomal formulations in the treatment of various ocular diseases [82]. Liposomes are good for ophthalmic drug delivery because they decrease rapid clearance from the vitreous cavity, enhance intravitreal half-life of drugs, and reduce toxicity associated with higher dosage [49].

### 11.5. Liposomes in Cancer Therapy

Cancer is a serious health problem in the world. Chemotherapeutic agents used for cancer treatment usually cause undesired side effects because of their harmful effects on normal, noncancerous tissues [76]. Additionally, cancer drugs do not reach all cancerous cells, which can lead to metastasis and drug resistance. Liposomes, on the other hand, can dispense large quantities of drugs to tumors while avoiding off targets and elimination by the mononuclear phagocyte system, making treatment more effective. Peptide functionalized liposomes can be used to target a number of selective receptors overexpressed on cancer cells [76]. As mentioned above, tumor cells tend to have a more acidic environment, which can be exploited for targeted drug delivery, using pH-sensitive liposomes. Doxil^®^ (or Caelyx^®^ in Europe) was the first FDA (Food and Drug Administration)-approved pegylated liposomal doxorubicin for the treatment of various cancers [41].

### 11.6. Liposomes as Delivery Systems for Antibiotics and Anti-Infectives

Multidrug resistance in bacteria is a crucial problem. It is explained by the use of various resistance mechanisms by the bacteria such as biofilms formation, reduced membrane permeability, antibiotic-modifying enzymes, and efflux pumps [83]. In light of this, liposomes are particularly useful to potentiate the activity of antibiotics and bypass resistance mechanisms, while reducing associated toxicity [84]. Previous studies have demonstrated that liposomal antibiotics have increased activity against various resistant bacteria. Indeed, Rukavina et al. developed azithromycin loaded liposomes that improved the antibiotic’s activity against skin infections caused by methicillin resistant *staphylococcus aureus*. The formulation also retained azithromycin in the skin more efficiently and prevented biofilm formation, with a minimum biofilm formation concentration 32 fold lower than the free drug [85]. In a recent study, Ribeiro and coworkers showed the improved in vitro antimicrobial activity of their hybrid liposomal formulation composed of pectin, liposomes and norfloxacin against multidrug resistant strains of *Pseudomonas aeruginosa*, *E. coli*, *Salmonella* sp., and *Campylobacter jejuni* [84]. The pectin was used for its resistance to low pH and its ability to interact with mucin. It is believed to increase the antibiotic’s bioavailability after oral administration. Additionally, an in vivo chicken embryo study confirmed the safety of the formulation. Finally, in the treatment of pulmonary *P. aeruginosa* infections in cystic fibrosis patients, inhaled liposomal tobramycin is the drug of choice. Many studies reported its efficacy against the pathogen, along with an enhanced bacterial killing at reduced doses [86]. Several liposomal anti-infective treatments have been approved for the treatment of various infections as shown in Table 2.

## 12. Conclusions

Liposomes have shown great promise in drug delivery, with several approved liposomal drugs on the market in different fields. However, they still have some limitations that need to be addressed such as toxicity issues, encapsulation efficiencies, stability, increased targeted delivery, and scale-up production. The restraints linked to the conventional techniques of liposomal production can be addressed by the use of the SuperLip method. The present review discussed the composition of liposomes, the various methods used for their production and characterization, as well as their applications in various fields such as cancer, infectious diseases, and eye and brain diseases.

## 13. Future Direction

Future studies should involve more research on tissue penetration and efficacy of liposomal drugs. Indeed, few studies have used Matrix-Assisted Laser Desorption/Ionization-Imaging Mass Spectrometry (MALDI-IMS) to examine the penetration of liposomes [41]. There should also be more efforts in the drug development process to increase drug release from liposomes to target sites to increase specificity. This will allow for a more effective targeted drug delivery in various fields (infectious diseases, cancer, vaccines, eye and brain diseases). New rapid, cost-effective, scalable manufacturing techniques with reduced steps for liposome production are greatly needed to improve scale-up production. Similar methods that aim to support or stabilize the aqueous layer of liposomes instead of the lipid layer should be explored in the future as they could improve drug loading and reduce leakage.

## Figures and Tables

**Table 1:** Advantages and disadvantages of liposomes preparation methods.

Methods	Advantages	Disadvantages
DRV/thin film hydration	Simple, high EE for lipophilic drugs	Use of organic solvent, low EE for hydrophilic drugs, MLV formed, heterogeneity, difficulty scaling up, size reduction required
RPE	Simple, high EE for hydrophilic drugs	Challenge removing organic solvent, LUV and MLV formed, low EE for lipophilic drugs, not suitable for fragile molecules, size reduction required
Injection	Simple, SUV formed in one step, good reproducibility	Heterogeneity, low EE, possible degradation of active drug, difficulty removing ethanol, time consuming
Emulsion	High EE, control over liposomes size	Use of organic solvent, LUV formed, multiple steps
Detergent removal	No organic solvent used, proteins encapsulation, size uniformity, good reproducibility	LUV formed, poor EE of hydrophobic drugs, detergent residues, time consuming
Sonication	Easy, formation of SUVs	Possible degradation of phospholipids, possible metallic pollution when using probe
Extrusion	Simple, size uniformity, SUV formation, good reproducibility	Use of high pressure, possible clogging of membrane, product loss, laborious, time consuming
Microfluidization	SUV formation in one step, continuous production, size uniformity, good EE [29]	Use of organic solvent, requires specific setup, high energy and pressure used, difficult large scale production, costly [30]
Freeze-drying	Storage stability, sterile liposomes, extended shelf-life	Applications might be limited when carbohydrates are used as cryo-protectants, low EE, potential damage to bilayer and size increase of liposomes due to membrane fusion [31,40]
Heating	No organic solvent used, sterile product [17], scalable	High temperature makes continuous manufacturing impractical, low EE, LUV formed, size reduction required
SuperLip	SUV formation in one step, continuous production, superior high EE, size uniformity, no organic solvent	Use of high pressure, more complex
Active encapsulation	High EE, stable retention of drug [39]	Only applies to amphipathic weak bases/acids, complex synthesis of some derivatives [16]

**Table 2:** Approved liposomal formulations used in various applications.

Product	Drug	Target Treatment	References
Doxil^®^	Doxorubicin	Breast and ovarian cancer	[7,65]
Marqibo^®^	Vincristine sulfate liposomes	Hodgkin’s lymphoma and leukemia	[66]
Arikayce^®^	Amikacin	MAC and PA infections	[67]
Lipoquin^®^	Ciprofloxacin	MAC infections	[68]
Pulmaquin^®^	Ciprofloxacin	PA lung infections	[68]
TOBI^®^	Tobramycin	PA infections	[69]
Cayston^®^	Aztreonam	Gram negative infections	[70]
Colobreathe^®^	Colistin	Gram negative infections	[71]
Ambisome^®^	Amphotericin B	Fungal infections	[72,73]
Exparel^®^	Bupivacaine	Pain management	[7]
Inflexal^®^	Inactivated hemagglutinin of influenza virus strains A and B	Influenza	[7]
Epaxal^®^	Formalin inactivated hepatitis A virus, strain RG-SB	Hepatitis A	[73]
Moderna COVID-19	mRNA 1273	Covid-19	[74,75]

## Data Availability

Not applicable.
